# Predictive Value of CT Perfusion in Hemorrhagic Transformation after Acute Ischemic Stroke: A Systematic Review and Meta-Analysis

**DOI:** 10.3390/brainsci13010156

**Published:** 2023-01-16

**Authors:** Jie Xu, Fangyu Dai, Binda Wang, Yiming Wang, Jiaqian Li, Lulan Pan, Jingjing Liu, Haipeng Liu, Songbin He

**Affiliations:** 1 Department of Neurology, Zhoushan Hospital, Wenzhou Medical University, Zhoushan 316000, China; 2 Research Centre for Intelligent Healthcare, Coventry University, Coventry CV1 5FB, UK

**Keywords:** acute ischemic stroke, hemorrhagic transformation, computed tomography perfusion, meta-analysis

## Abstract

Background: Existing studies indicate that some computed tomography perfusion (CTP) parameters may predict hemorrhagic transformation (HT) after acute ischemic stroke (AIS), but there is an inconsistency in the conclusions alongside a lack of comprehensive comparison. Objective: To comprehensively evaluate the predictive value of CTP parameters in HT after AIS. Data sources: A systematical literature review of existing studies was conducted up to 1st October 2022 in six mainstream databases that included original data on the CTP parameters of HT and non-HT groups or on the diagnostic performance of relative cerebral blood flow (rCBF), relative permeability-surface area product (rPS), or relative cerebral blood volume (rCBV) in patients with AIS that completed CTP within 24 h of onset. Data Synthesis: Eighteen observational studies were included. HT and non-HT groups had statistically significant differences in CBF, CBV, PS, rCBF, rCBV, and rPS (*p* < 0.05 for all). The hierarchical summary receiver operating characteristic (HSROC) revealed that rCBF (area under the curve (AUC) = 0.9), rPS (AUC = 0.89), and rCBV (AUC = 0.85) had moderate diagnostic performances in predicting HT. The pooled sensitivity and specificity of rCBF were 0.85 (95% CI, 0.75–0.91) and 0.83 (95% CI, 0.63–0.94), respectively. Conclusions: rCBF, rPS, and rCBV had moderate diagnostic performances in predicting HT, and rCBF had the best pooled sensitivity and specificity.

## 1. Introduction

Stroke is a major cause of death and disability around the world. The absolute number of stroke deaths increased by 43% from 1990 to 2019 and that of disability-adjusted life years (DALYs) lost rose by 32% [1]. Acute ischemic stroke (AIS) is the commonest type of stroke, and its poor prognosis is closely related to hemorrhagic transformation (HT). HT is part of the natural history of AIS and one of its major complications, with a spontaneous conversion rate of 7–29% [2], and in patients treated with acute reperfusion stroke, the incidence can be over 40% [3], resulting in a mortality of 3% [4]. The mechanism of HT is not fully understood. At present, it is known that the etiology of stroke, the age of the disease, the use of thrombolytic drugs, and other factors are related to HT [5], wherein focal hemodynamic changes may play a key role. Some scores have been proposed to predict the risk of HT, such as the hemorrhagic transformation index (HTI) [6] and the Ischemic Stroke Predictive Risk Score (ISCORE) [7]. HT after AIS is still an open topic without any established tool that can achieve accurate prediction.

Computed tomography perfusion (CTP) can quantitatively reflect cerebral blood flow and hemodynamic information and has been widely used in the hemodynamic evaluation of AIS. Many clinical observations commonly suggest that admission CTP parameters can be significantly different between HT and non-HT groups, providing the possibility of HT prediction [8,9,10,11,12,13,14,15,16,17,18,19,20,21,22,23,24,25,26,27,28]. Souza et al. suggested that admission CTP parameters were independent predictors of HT in AIS [24]. There is no consistent conclusion on the predictive value of various CTP-derived indicators. Jain et al. found that relative cerebral blood volume (rCBV) could predict HT with a specificity of 72%, whilst there was no difference in relative cerebral blood volume (rCBF) between HT and non-HT groups [16]. Li et al. also found no significant difference in rCBV between these two groups [11]. Langel et al. showed that a rCBF < 4.5% was the best predictor of HT (with a sensitivity of 71.0% and specificity of 52.5%) [18]. Although CTP may be used for predicting HT after AIS, it is still undetermined which parameters are most efficient in differentiating HT and non-HT patients and predicting HT. Some hemodynamic thresholds have been established to evaluate the severity of AIS, for example, regions of the time-to-peak of the residual function (Tmax) > 10 sec stand for severe hypoperfusion regions [29], and a Tmax > 6 s and rCBF < 30% are important indicators to evaluate the penumbra and core infarction area in AIS patients [30,31]. However, there is a lack of standards for predicting HT among AIS patients.

To fill this gap, a systematic literature review of relevant studies was conducted to find out if admission CTP parameters are significantly different between HT and non-HT groups. Based on the results of 18 observational studies [8,9,10,11,12,13,14,15,16,17,18,19,20,21,22,23,24,25,26,27,28], the predictive value of perfusion parameters within 24 h of AIS in predicting HT was evaluated. Among the studies, CTP imaging was performed within 24 h of onset, before any reperfusion therapy. Despite the inconsistency among these studies in CTP parameters, some CTP parameters were found to be potentially HT-relevant. The results provide a reference for future works aimed toward CTP-enhanced HT prediction.

## 2. Materials and Methods

This meta-analysis was performed in accordance with the Preferred Reporting Items for Systematic Reviews and Meta-Analysis (PRISMA) guidelines [32].

### 2.1. Eligibility Criteria

Types of studies: Prospective or retrospective observational studies on the predictive value of CTP parameters in patients with AIS within 24 h. No CTP parameters or publication date restrictions were imposed. When it came to the diagnostic performance of CTP parameters for HT, only the performance of relative parameters was reported. The differences between studies in post-processing software are still impossible to eliminate [33], and the individual differences in pathophysiology are unavoidable.

Types of participants: Patients with AIS of any age were considered to be included in the study. For all patients, CTP was performed within 24 h of onset.

Intervention: The studies involved in this meta-analysis were observational and did not interfere with any clinical intervention except for clinical decision-making.

Outcome measurement: The only outcome was HT. HT was defined as hemorrhagic infarction (HI1 and HI2) or parenchymal hematoma (PH1 and PH2) according to the European Cooperative Acute Stroke Study (ECASS II) [34].

### 2.2. Information Sources

Studies were identified by searching 6 mainstream electronic databases, including PubMed, Cochrane, Embase, VIP, Wanfang, and the Chinese Biomedical Literature Database. The retrieval time was from the establishment of the database to 1st October 2022. The languages were restricted to English and Chinese. 

The search terms used for all the databases were as follows: acute ischemic stroke, computed tomography perfusion, and hemorrhagic transformation.

Search strategy: PubMed

01. acute ischemic stroke. ti, ab.

02. acute stroke. ti, ab.

03. cerebral ischemia. ti, ab.

04. cerebral infarction. ti, ab.

05. cerebrovascular ischemia. ti, ab.

06. ischemic stroke. ti, ab.

07. stroke. ti, ab.

08. 01 or 02 or 03 or 04 0r 05 or 07

09. computed tomography perfusion. ti, ab.

10. CT perfusion. ti, ab.

11. perfusion computed tomography. ti, ab.

12. perfusion CT. ti, ab.

13. 09 or 10 or 11 or 12

14. hemorrhagic transformation. ti, ab.

15. cerebral hemorrhage. ti, ab.

16. parenchymal hematoma. ti, ab.

17. hemorrhage conversion. ti, ab.

18. intracerebral hemorrhage. ti, ab.

19.14 or 15 or 16 or 17 or 18

20. 08 and 13 and 19

### 2.3. Study Selection

The eligibility assessment was performed independently by 2 reviewers. Firstly, for multiple papers regarding the same study, only the most detailed one was included. Secondly, original studies without AIS patients and other publication types (case reports, meeting abstracts, review articles, etc.) were excluded. Finally, the remaining studies that did not provide the original data of CTP parameters of a normal distribution or the diagnostic performance of rPS, rCBF or rCBV were excluded. The qualification evaluation process was identical for all the publications.

### 2.4. Data Collection

Information on the following was extracted from each included study: author; publication time; characteristics of patients (including age and sex); inclusion and exclusion criteria of the trial; latest cut-off time from onset to perform CTP; follow-up imaging; CT parameter value; result measurement (HT); the number of HT subtypes classified by the ECASS II; sufficient data for the reconstruction of 2 × 2 tables for the determination of the diagnostic performance of relative permeability-surface area product (rPS), rCBF and rCBV for prediction of HT in AIS (cutoff, true positive [TP], false positive [FP], true negative [FN], and true negative [TN] values). The details were extracted from the included studies by one co-author and double-checked by another with differences resolved through discussion.

### 2.5. Quality Evaluation

The quality evaluation was carried out independently by the two authors. If there was any difference, more authors were involved to discuss and finalize the evaluation results. The Newcastle-Ottawa Quality Assessment Scale (NOS) was used to evaluate the quality of all the original data on CTP parameters. The NOS is a semi-quantitative evaluation scale with a range of 0–9, with <5 considered high bias risk, 5–7 medium bias risk, and 8–9 low bias risk. The Quality Assessment of Diagnostic Accuracy Studies-2 (QUADAS-2) was performed to evaluate the quality of studies that provided information on the diagnostic performance of rPS, rCBF, or rCBV. The QUADAS-2 scale includes 14 items, mainly the patient selection, index test, reference standard, flow and timing, and applicability. Finally, bias could be defined as “high”, “low”, or “uncertain”.

### 2.6. Statistical Analysis

The meta-analysis of the included studies that provided original CTP parameters was carried out with RevMan software, version 5.4 (Cochrane Collaboration, Oxford, UK). An odds ratio (OR) was used between counting data, while mean difference (MD) was used for combined analysis between measurement data. The 95% CI was used to represent the combined effect. An I^2^ test was performed to determine whether there was any heterogeneity among the studies. The homogeneity among studies was defined as *p* > 0.1, I^2^ < 50%, where the fixed effect model was used for meta-analysis. Accordingly, heterogeneity was defined as *p* < 0.1, I^2^ > 50%, where the random effect model was used. Forest plots were drawn using RevMan software, which has been commonly used in meta-analyses. A forest plot summarizes the information of pooled studies, visualizes the heterogeneity, and shows the estimated common effect, I^2^.

The diagnostic meta-analysis of the included studies that provided the diagnostic performance of rPS, rCBF, or rCBV was performed using Stata, version 15.0 (StataCorp LP, College Station, TX, USA). The bivariate random effect model was used to calculate the sensitivity, specificity, and 95% CI of the combination. The hierarchical summary receiver operating characteristic (HSROC) was drawn and the area under the curve (AUC) was calculated. Cochrane-Q and I^2^ tests with *p* < 0.05 and I^2^ >50 indicated the presence of heterogeneity, respectively. Deeks’ funnel plots were drawn using Stata. Deeks’ funnel plots have been widely used for evaluating publication bias, such that the graph shows a symmetrical inverted funnel shape when there is no bias among the studies [35]. *p* < 0.1 was considered statistically significant. 

Meta-regression was conducted according to the following covariates: country (home or abroad); study design (prospective or retrospective); reference standard (only non-contrast CT (NCCT) was used as follow-up image or not); and publication time (published in the last 7 years or not). All categorical covariates were coded in a binary manner. *p* < 0.05 was considered statistically significant.

## 3. Results

### 3.1. Study Selection

**Figure 1:**
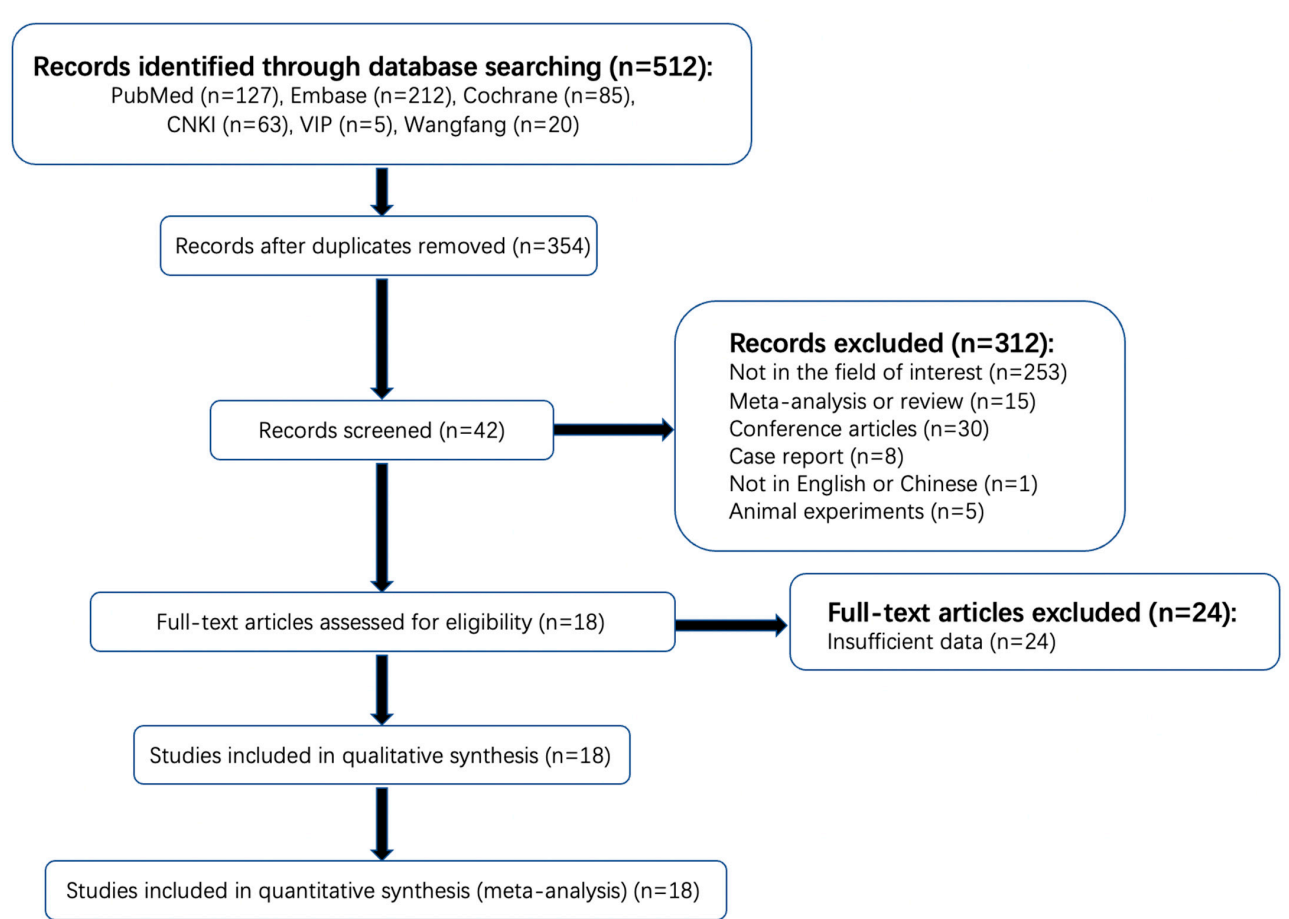
Flowchart of search strategy and selection of reports.

The detailed literature selection process is shown in Figure 1. A total of 512 articles were found in a systematic search. One hundred and fifty-eight duplicated articles were excluded on Endnote software, version 20.1 (Thomson Reuters, Philadelphia, PA, USA). Among the remaining 354 publications, 253 items were not in the area of interest and therefore excluded after reading the titles and abstract (the studies of patients with AIS were not included or did not complete CTP within the time limit or the outcome index of observation did not include HT). Thirty conference abstracts, fifteen reviews, eight case reports, five animal experiments, and one study of non-Chinese or -English literature were also excluded. Then, a full-text review of the remaining 42 potentially qualified articles was conducted. Twenty-four articles that did not provide the original data of CTP parameters of a normal distribution or the diagnostic performance of rPS, rCBF, or rCBV were further excluded. Finally, 18 articles were included, with a total sample size of 1423 cases, of which 13 articles provided the original data of CTP parameters that followed a normal distribution, with a sample size of 831 cases. A total of 11 articles provided sufficient information for the determination of the diagnostic performance of rPS, rCBF, or rCBV, with a sample size of 906 patients. No unpublished literature was included.

### 3.2. Study Characteristics

The features of the included study are listed in Table 1, Table 2 and Table 3. All 18 articles included observational experiments, of which 8 and 10 were prospective and retrospective studies, respectively. Among these studies, the longest follow-up time was seven days. The CTP examinations were completed within 24 h after onset. Follow-up CT or MRI imaging examination was performed to evaluate the occurrence of HT. No intervention was carried out for the patients, except for standardized clinical medical decision-making. HT was the only outcome index, and there was no distinction between the types of HT.

Of the 14 studies reporting CTP parameters, 10 used CT scanners with 64 or more slices, 3 used 16-slice CT scanners, and 1 used 8-slice CT scanners. In terms of follow-up imaging, three studies used CT or MRI as the reference standard for HT. The reported treatments included intravenous thrombolysis, arterial thrombectomy, arterial thrombectomy based on thrombolysis, and no reperfusion therapy.

Only six studies classified HT according to the ECASS II classification [9,15,16,17,19,23]. Among these studies, one study did not include HI1 subtype [15] and another did not include HI1 and HI2 subtypes [23] because the researchers consider that the HI1 subtype has low clinical relevance for decision-making, and the remaining studies included four classifications of HT as defined by the ECASS II [8,9,10,11,12,13,14,16,17,18,19,20,21,22,24,25,26,27,28].

### 3.3. Quality Evaluation

**Figure 2:**
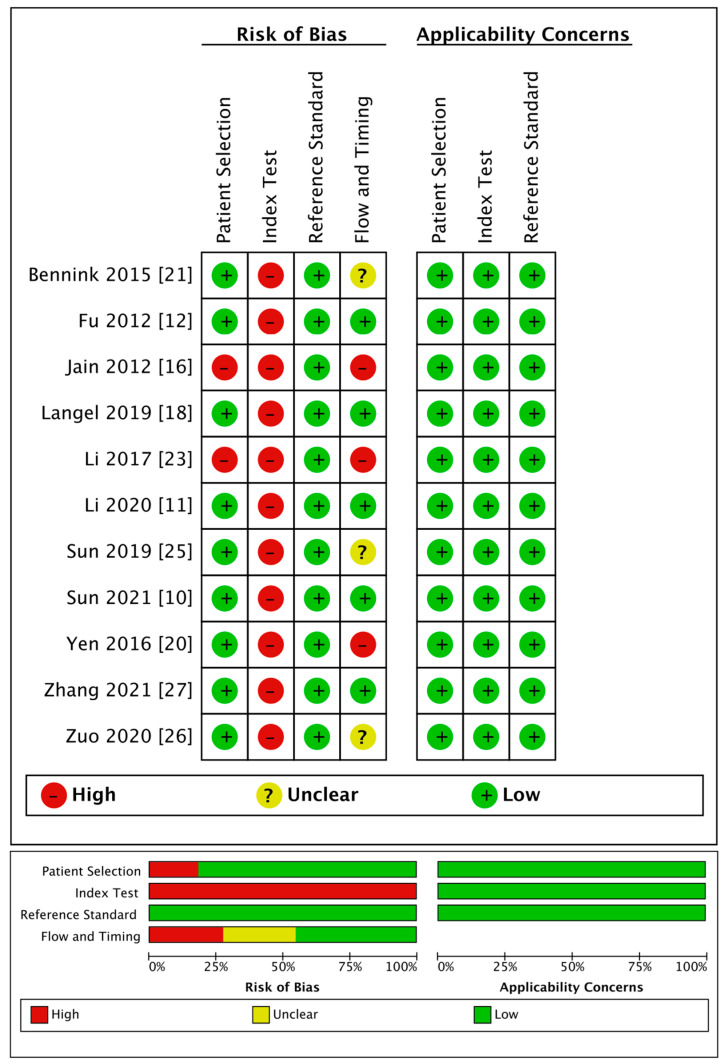
Quality of the 11 included studies that provided diagnostic performance of rPS, rCBF, or rCBV.

The detailed quality evaluation results are shown in Table 4 and Figure 2. The NOS results indicated a low bias risk in the included publications, including six articles with eight points (i.e., low bias risk); six articles with seven points, and only one article with six points (i.e., medium bias risk).

The QUADAS-2 evaluation showed medium to high quality generally except for the index tests. Regarding patient selection, two articles used a case-control design, resulting in a high risk of bias. As for the index tests, because the thresholds used for diagnosis in all the articles were obtained through statistical analyses, there was no pre-set threshold, which led to a high risk of bias. Regarding the reference standards, all the trials included were regarded as having a low risk of bias. Regarding the flow and timing, three studies were regarded as having a high risk of bias because they did not include all the cases, and four studies had an unclear risk of bias as they did not mention the follow-up time. All studies had high clinical applicability.

### 3.4. Meta-Analysis Results

#### 3.4.1. Differences in CTP Parameter Values between HT and non-HT Groups

**Figure 3:**
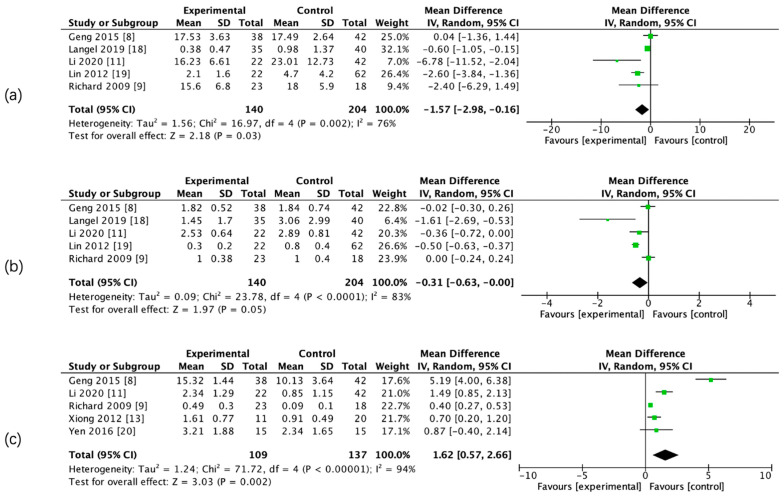
Forest maps of absolute computed tomography perfusion (CTP) parameters for hemorrhagic transformation (HT) and non-HT groups. CBF (**a**), CBV (**b**), and PS (**c**).

**Figure 4:**
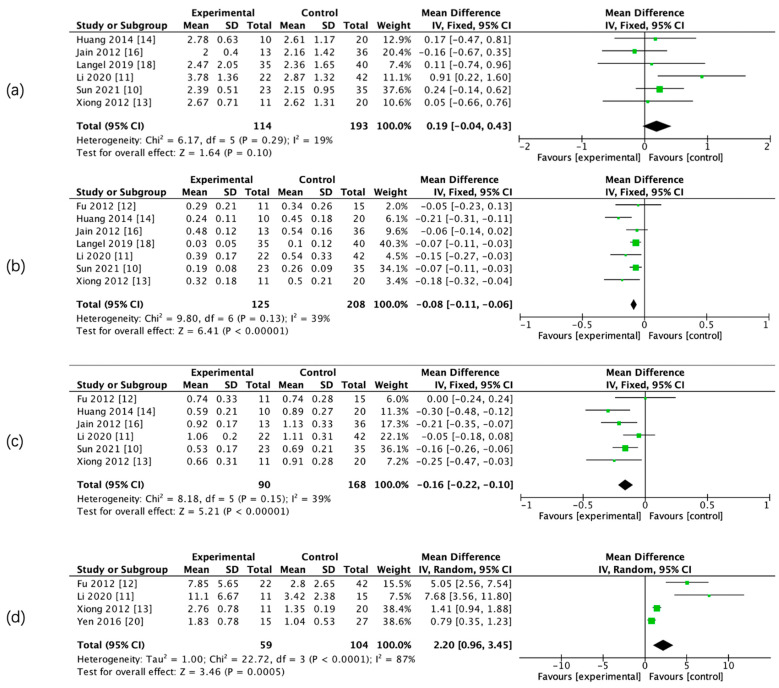
Forest maps of relative CTP parameters for HT and non-HT groups. Relative mean transit time (rMTT) (**a**), rCBF (**b**), rCBV (**c**), and rPS (**d**).

The results of the meta-analysis showed that there was a significant difference in admission CBF, CBV, PS, rCBF, rCBV, and rPS parameters between the two groups, whereas there was no significant difference in admission relative mean transit time (rMTT) between the two groups. (Figure 3 and Figure 4, Table 5).

#### 3.4.2. Recombinant Tissue Plasminogen Activator (rt-PA) Usage

**Figure 5:**
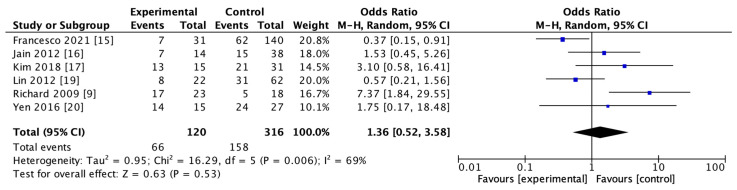
Forest map of recombinant tissue plasminogen activator (rt-PA) usage in HT and non-HT groups.

The use of rt-PA for reperfusion therapy at admission was reported in 6 studies including 436 subjects in both HT and non-HT groups. The results of the heterogeneity study suggested that there was heterogeneity among the studies (I^2^ = 69%, *p* = 0.006). The results of the meta-analysis using the random effect model showed that there was no significant difference in the use of rt-PA between the two groups (OR = 1.36; 95% CI, 0.52–3.58–0.53). The details are shown in Figure 5.

#### 3.4.3. The Predictive Performance of rPS, rCBF, and rCBV for HT

**Figure 6:**
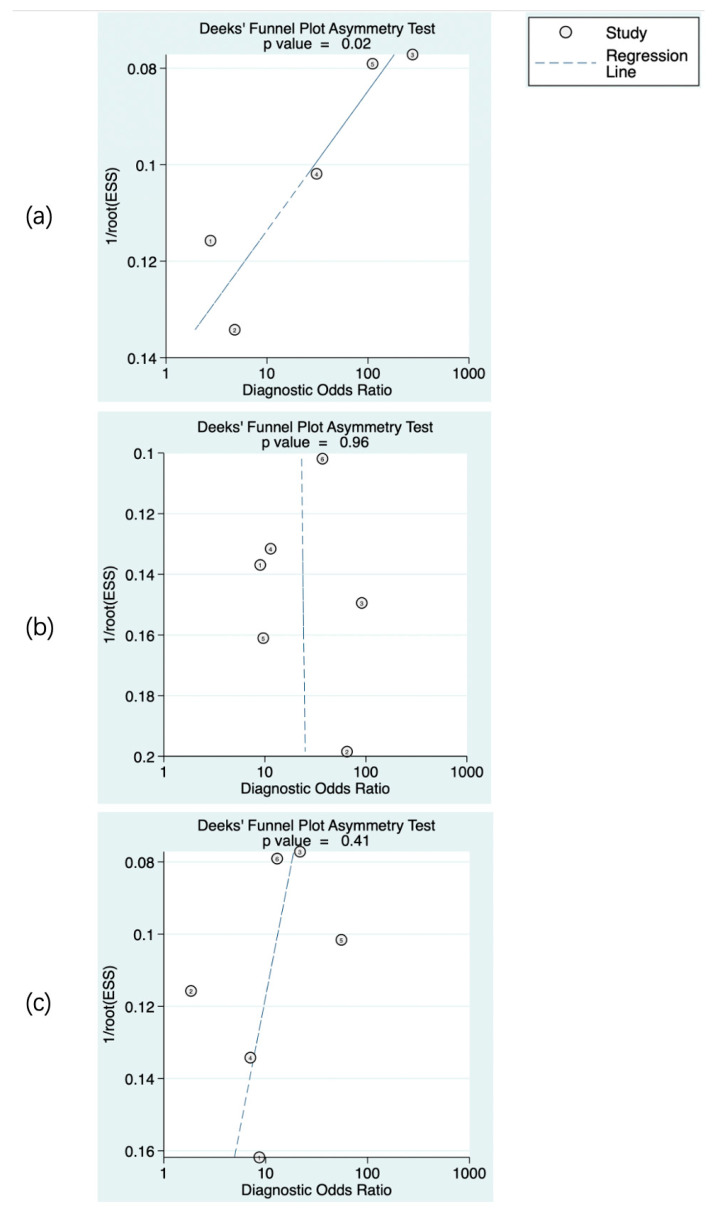
Publication bias of the studies included. rCBF (**a**), rPS (**b**), and rCBV (**c**). The numbers in the figures denote the data from different studies.

**Figure 7:**
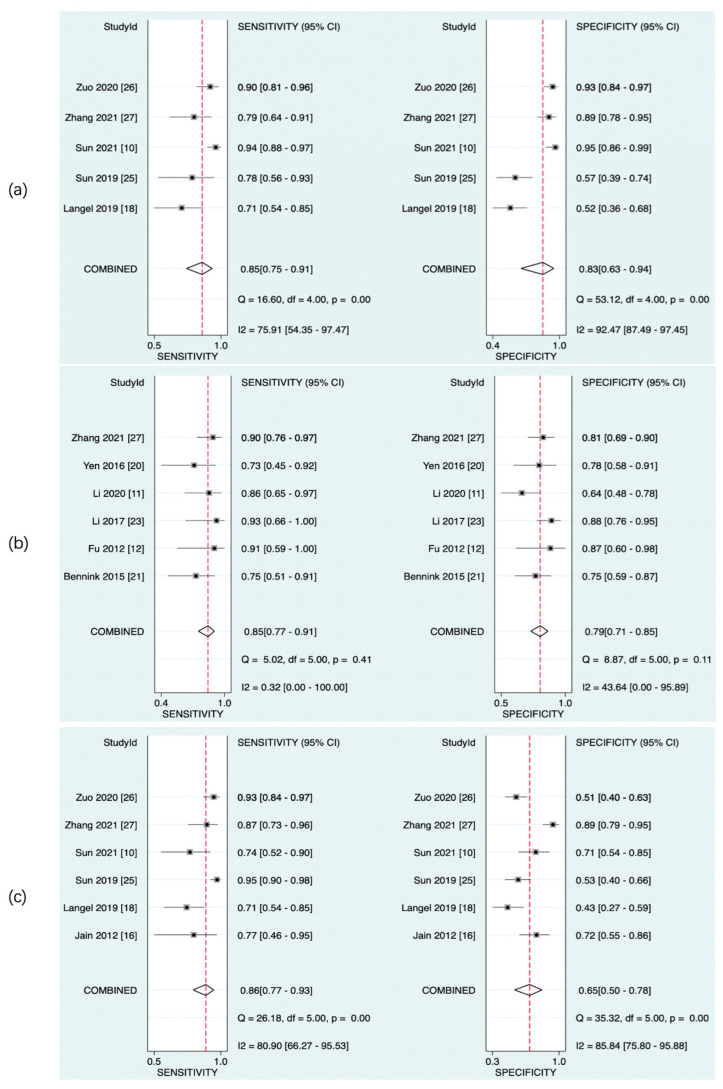
Pooled sensitivity and specificity of CTP parameters for diagnosis of HT. rCBF (**a**), rPS (**b**), and rCBV (**c**).

**Figure 8:**
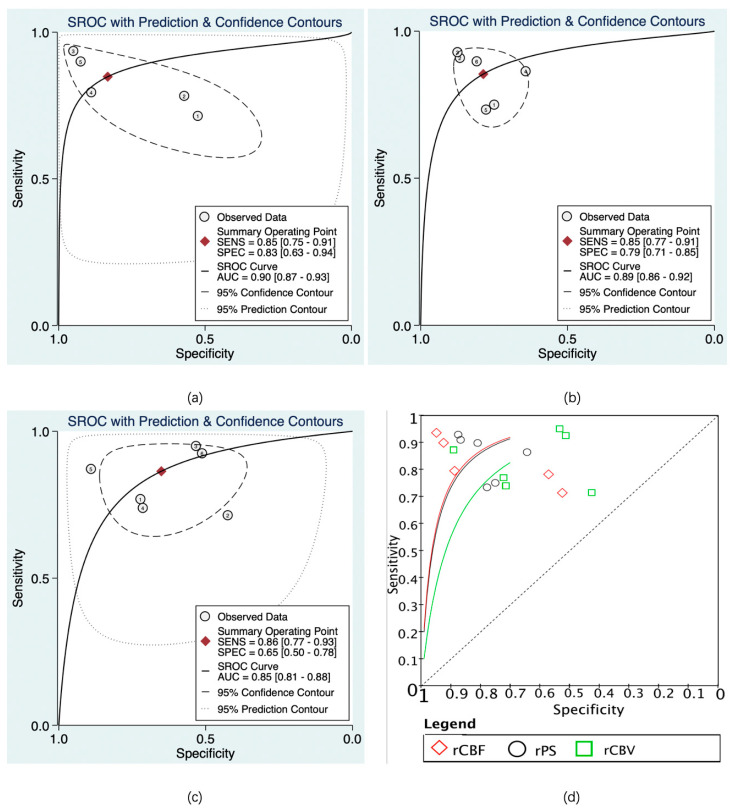
Summary receiver operating characteristic (SROC) of CTP parameters for diagnosis of HT. rCBF (**a**), rPS (**b**), rCBV (**c**), and SROC of rCBF, rPS, and rCBV (**d**). The numbers in the circles denote the data from different studies.

The predictive value of rCBF for HT was reported in 5 studies with a total of 595 patients. The Deeks’ funnel plot suggested a high likelihood of publication bias (*p* = 0.02). The bivariate random effect model indicated that the pooled sensitivity and specificity of rCBF for predicting HT were 85% (95% CI, 75–91%) and 83% (95% CI, 63–94%), respectively. The positive likelihood ratio was 5.1 (95% CI, 1.9–13.5). The negative likelihood ratio was 0.18 (95% CI, 0.09–0.37). The DOR was 28 (95% CI, 5–143). The AUC of HSROC was 0.90 (95% CI, 0.87–0.93). The Cochrane-Q and I^2^ tests showed that there was heterogeneity in sensitivity (Q = 16.6, *p* = 0.00; I^2^ = 75.91) and specificity (Q = 53.12, *p* = 0.00; I^2^ = 92.47) (Figure 6a,Figure 7a and Figure 8a).

The predictive value of rPS for HT was reported in 6 studies with a total of 364 patients. The Deeks’ funnel plot suggested a low likelihood of publication bias (*p* = 0.96). The bivariate random effect model indicated that the pooled sensitivity, specificity of rPS for predicting HT were 85% (95% CI, 77–91%) and 79% (95% CI, 71–85%), respectively. The positive likelihood ratio was 4 (95% CI, 2.8–5.6). The negative likelihood ratio was 0.19 (95% CI, 0.11–0.30). The DOR was 22 (95% CI, 10–45). The AUC of HSROC was 0.89 (95% CI, 0.86–0.92). Cochrane-Q and I^2^ tests showed that there was no heterogeneity in sensitivity (Q = 5.02, *p* = 0.41; I^2^ = 0.32) and specificity (Q = 8.87, *p* = 0.11; I^2^ = 43.64) (Figure 6b, Figure 7b and Figure 8b).

The predictive value of rCBV in HT was reported in 6 studies with a total of 645 patients. The Deeks’ funnel plot suggested a low likelihood of publication bias (*p* = 0.41). The bivariate random effect model indicated that the pooled sensitivity and specificity of rCBF for predicting HT were 86% (95% CI, 77–93%) and 65% (95% CI, 50–78%), respectively. The positive likelihood ratio was 2.5 (95% CI, 1.6–3.7). The negative likelihood ratio was 0.21 (95% CI, 0.12–0.38). The DOR was 12 (95% CI, 5–28). The AUC of HSROC was 0.85 (95% CI, 0.81–0.88). The Cochrane-Q and I^2^ tests showed that there was heterogeneity in sensitivity (Q = 26.18, *p* = 0.00; I^2^ = 80.90) and specificity (Q = 35.32, *p* = 0.00; I^2^ = 85.84) (Figure 6c, Figure 7c and Figure 8c).

#### 3.4.4. Meta-Regression

**Figure 9:**
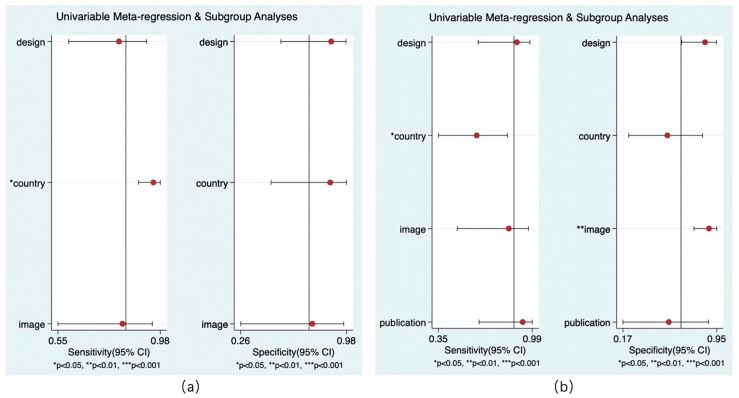
Results of meta-regression. rCBF (**a**) and rCBV (**b**).

Only the models for country and reference standard resulted in covariates remaining significant. Whether the study was performed in China had an impact on the sensitivity of rCBF (*p* < 0.05) and the sensitivity of rCBV (*p* < 0.01) to predict HT. Whether the NCCT was the only follow-up image was related to the specificity of rCBV in the diagnosis of HT (*p* < 0.01). No covariates were found for the subgroup analysis (Figure 9).

## 4. Discussion

### 4.1. Summary of Findings

In this systematic review and meta-analysis, all the possibly relevant studies were included, involving 1423 patients with AIS. In the HT group, the values of CBF, CBV, rCBF and rCBV were lower, while the PS and rPS were higher compared with the non-HT group. There was no significant difference in the value of rMTT and the use of rt-PA between the two groups. The diagnostic meta-analysis showed that rCBF, rPS, and rCBV had moderate diagnostic performances in the prediction of HT in AIS. RCBF had the best pooled sensitivity and specificity, followed by rPS and rCBV. The results commonly suggested that the CTP parameters could predict the risk of HT in patients with AIS.

### 4.2. Advantages of CTP and Its Potential for Detecting HT

CTP has been widely used in patients with AIS and can evaluate ischemic penumbra visually [36,37]. CTP has the advantages of relative rapidity and wide applicability [38,39]. In addition to CTP, some studies have reported the diagnostic value of magnetic resonance imaging (MRI) in AIS [40,41,42,43]. Compared with CTP, MRI might provide more precise estimates of BBB disruption [44] and have a higher diagnostic value [45]. In terms of HT detection, it has been suggested that the T2 gradient-recalled echo sequence of MRI is the most reproducible method for detecting HT and has a higher sensitivity than CT [46], but MRI is less applicable due to contraindications, higher prices, lower availability, especially in low-resource areas, and certain limitations (metal implants, cardiac pacemaker implants, high fever, etc.). For a time-dependent disease such as AIS, reasonable clinical decisions should be made as soon as possible, whereby CTP is still a preferable option [15]. Current guidelines [47] also recommend the use of CTP for selecting mechanical thrombectomy AIS patients who have large vessel occlusion (LVO) in the anterior circulation within 6–24 h of the last known normal. A recent meta-analysis including 505 individuals showed that patients benefited from mechanical thrombectomies guided by CTP parameters in the extended time window [48]. CTP-enhanced HT prediction could potentially improve the current clinical practice in the diagnosis and management of AIS.

CTP provides rich hemodynamic information that is not limited to the evaluation of ischemic penumbra. The CTP parameters rPS, rCBF, and rCBV showed moderate predictive power for HT after AIS, with the AUC of SROC ≥ 0.8 for all. At present, many studies suggest that the disruption of the BBB and hypoperfusion could significantly contribute to the development of brain injury and HT [49,50]. The rPS reflects the difference in cerebral vascular permeability between the affected side and the healthy side, which indicates the severity of BBB damage [51]. The rCBF and rCBV can reflect the extent of CBF and CBV reduction in the infarct area, indicating the severity of hypoperfusion, which is an inherent characteristic of AIS [52]. The CTP parameters might reflect the early hemodynamic changes of HT. However, HT is a complicated process that involves multiple pathophysiologic factors, of which many are undetectable from CTP imaging. In addition, the information lost is inevitable in the post-processing of CTP images [53]. CTP could be integrated with other clinical information to achieve a more individualized, precise, and stable multi-parameter diagnostic framework for predicting HT [54,55,56].

Current studies on CTP mainly focus on patients with anterior circulation cerebral infarction. Until recently, perfusion imaging often allowed the examination of brain perfusion only in a predefined tissue block, encompassing only a supratentorial brain slice of, e.g., 4 cm thickness, depending on the manufacturer, which provides little information on vertebrobasilar circulation [57]. There is a small sample study indicating that CTP imaging parameters may contain prognostic information on functional outcomes in patients with AIS due to basilar artery occlusion [58]. The correlation between CTP and HT in the vertebrobasilar territory deserves further study.

Researchers have proposed new hemodynamic thresholds regarding the prediction effect of CTP on HT, but the thresholds for predicting HT were also inconsistent [8,9,10,11,12,13,14,15,16,17,18,19,20,21,22,23,24,25,26,27,28]. Therefore, the optimal thresholds for predicting HT could not be obtained quantitatively through a meta-analysis but are worth further exploration in the future.

### 4.3. Heterogeneity among Studies: Comparison of Absolute and Relative Parameters of CTP

Concerning the clinical application of CTP, it is necessary to note the heterogeneity among different studies. The CBF, CBV, and PS values were subject to study heterogeneity, while the relative indicators showed lower heterogeneity. We tried to eliminate some of the literature and carried out a subgroup analysis according to country, follow-up imaging, study design, and other factors, but still could not eliminate the heterogeneity of all factors. Different methods of assessing CT perfusion, post-processing, and treatment can lead to heterogeneity. The differences in post-processing software of different image systems are still impossible to eliminate [33], and the individual differences in pathophysiology and cerebral perfusion among patients might lead to change in the absolute parameters of CTP. Some researchers including Langel et al. [18] and Jain et al. [16] recommended the use of relative indicators of CTP. The relative index represents the absolute value of a brain region divided by the value of the contralateral brain region of the parameter. This partially eliminates the differences between various devices and patients, making the results more robust against individual differences. In addition, Xiong et al. used Spearman’s rank correlation to study the correlation between CTP parameters and HT. They found that there was a higher correlation between rPS and HT than PS (r = 0.496 vs. r = 0.821) [13]. Therefore, although differences were observed in the CBF, CBV, rCBF, rCBV, PS, and rPS values between the HT and non-HT groups, only the diagnostic performance of the rCBF, rCBV, and rPS values for HT was reported. 

### 4.4. Pathophysiological Mechanisms of HT after AIS

The results suggested that high BBB permeability and hypoperfusion status indicated by CTP parameters were associated with HT. Studies have shown that the main mechanisms related to HT include hyperactive ischemic cascades with increased matrix metalloproteinase (MMP) levels [59,60], reperfusion injury [61,62,63], BBB destruction [64,65,66], and blood coagulation disorder [67]. As aforementioned, in addition to spontaneous HT, the use of rt-PA or endovascular treatment bears the risk of reperfusion HT, which depends on the type of reperfusion therapy [68,69,70,71]. A study indicated that the correlation between increased BBB values and HT was stronger in patients receiving endovascular thrombectomy (EVT) than in patients receiving rt-PA [17]. The adjustments based on treatment interference (e.g., IVT, EVT, etc.) might enable a fine-grained analysis and provide more detailed guidance for clinical practice. Langel et al. conducted an analysis on the prognosis of AIS patients who had undergone CTP imaging and were treated with IVT, wherein the results showed that CTP had a good predictive effect on HT [18]. However, some studies that evaluated the predictive value of CTP parameters for predicting HT were not grouped by treatment modality [20,25], which can be improved in future studies.

HT after the use of thrombolytic drugs might involve more complex biological processes [72]. Rt-PA could trigger harmful cascades, such as augmented neurovascular cell toxicity, which stimulates the generation of free radicals and results in cerebral cell death in the central nervous system [67,72]. The toll-like receptor 4 (TLR4) signaling pathway might be associated with inflammation and the exacerbation of post-ischemic brain damage. A recent study demonstrated that the TLR4 pathway was an important mechanism of rt-PA-induced HT, very likely via increasing MMP-9 expression [73]. Rt-PA-induced inflammation also plays an important role in the progression of HT in AIS [74]. Although more related mechanisms of thrombolytic drugs leading to HT are still to be explored, there is almost a consensus on the use of thrombolytic drugs increasing the risk of HT [75]. Interestingly, the meta-analysis of the use of thrombolytic drugs in HT and non-HT groups showed that there was no significant difference between the two groups (OR = 1.36, 95% CI, 0.52–3.58, *p* = 0.53). This conclusion should not be interpreted as the use of rt-PA having nothing to do with HT, but instead may be related to the small sample size (436 in total). The existing results indicate that thrombolysis might have an effect on the development of HT [76], which is not significant, possibly due to the small sample size. Meanwhile, it might also suggest that a more rational selection of patients for thrombolytic drugs may not increase the risk of HT in a later stage.

The analysis of rCBF and rCBV suggests that the blood flow per unit time and blood volume in the infarcted area, referring to the contralateral side, decreased more significantly in the HT group compared with the non-HT group. As previous studies have shown, cerebral hypoperfusion, which leads to oxygen and energy deficiency, might be a main etiology of brain injury and HT in AIS [77]. Cerebral hypoperfusion could induce the damage-associated molecular pattern (DAMP)/TLR4/NF-κB pathway [78] and down-regulate caveolin-1 [79], thereby upregulating the expression and activation of MMPs, especially MMP-9 and MMP-2 [56]. The BBB is mainly formed by cerebral endothelial cells (CECs) through tight junctions (TJs) [80]. MMPs could strongly degrade TJs and the basement membrane [81,82], aggravate BBB injury, and increase the possibility of HT. In addition, hypoxia also increases MMP9 activity, ultimately inducing more severe brain damage [54]. At the same time, a more severe hypoperfusion status might reflect collateral inadequacy [83,84]. In patients with severe middle cerebral artery (MCA) stenosis, distal cerebral perfusion may be more dependent on collateral circulation [85]. A retrospective cohort study showed that in AIS patients with MCA occlusion, those with poor collateral circulation were at high risk for HT [86]. In patients with other types of AIS, the role of collateral circulation needs further investigation.

The rPS could evaluate the relative rate of extravasation of contrast media through a ruptured BBB on the infarcted side compared with the contralateral side. The rPS results indicated that the relative rate of extravasation increased in the HT group with more significant damage of the BBB. The BBB is composed of brain endothelial cells (BECs), a basement membrane, astrocytes, and pericytes [87]. The BBB is not only a structural shelter between the central nervous system and periphery but also a metabolic and dynamic interface [88]. At present, destruction of the BBB is often considered an etiology of HT [89]. Under physiological conditions, BECs and their TJs maintain BBB permeability homeostasis. When AIS occurs, many mechanisms could disrupt the BBB and eventually lead to HT. For example, neuroinflammation stimulated by DAMP has been found to strongly contribute to disruption of the BBB [74]. MMP-9 could accelerate the migration and degradation of TJs and increase BECs’ endocytosis, thereby aggravating BBB damage [59]. After the BBB is destructed, blood-borne monocytes could infiltrate the brain and differentiate into macrophages, exacerbating the neuroinflammatory injury, thereby aggravating the propensity for HT [77]. In accordance with existing pathophysiological studies, our meta-analysis results highlighted the role of BBB destruction in HT.

## 5. Limitations and Future Directions

There are some limitations in this study. First of all, the number of studies on the predictive value of CTP in HT was limited. Some studies did not provide the original data of CTP parameters with a normal distribution or of the diagnostic performance of rPS, rCBF, or rCBV. Therefore, the number of patients included in the systematic review was small. Given the small sample size and insufficient raw data, the classification of HT was not performed. Secondly, because of the influence of mixed factors, such as examination equipment, post-processing algorithm, ethnic differences between patients, and other confounding factors that could not be summarized, some results were essentially heterogeneous. When evaluating the predictive value of rCBF for HT, the Deeks’ funnel plot suggested a high likelihood of publication bias. Therefore, more future research and standardized methodologies are needed to reduce bias. This study is hampered, among others, by different modes of follow-up imaging (CT or MRI), different or unknown (six studies) time points of follow-up imaging, and different levels of parametric thresholds. Thirdly, the CTP parameters of the included studies were different, and the thresholds of rPS, rCBF, and rCBV for predicting HT were also inconsistent, and the optimal threshold for predicting HT could not be obtained quantitatively through meta-analysis. Current studies on CTP mainly focus on patients with anterior circulation cerebral infarction, whereas perfusion imaging provides little information on vertebrobasilar circulation. Fourthly, ten of the eighteen included studies are from China, which might have introduced some bias, as East Asian populations may present with higher rates of HT [90].

A recent study exploring MRI and machine learning prediction models for predicting HT in AIS patients has shown that combining clinical variables, such as blood pressure and glucose, with radiomics features improved the prediction performance [91]. A logical next step for future research could be to establish a more individualized, precise, and stable multi-parameter diagnostic model for predicting HT with different etiologies, infarct sites, treatment approaches, treatment outcomes, and other factors. The classification of HT was not performed, which deserves more future research based on larger datasets. Although rCBF, rPS, and rCBV were considered to have moderate diagnostic performances in the prediction of HT in the literature, larger multicenter clinical trials should be carried out to verify the conclusion, eliminate regional and ethnic bias, and promote relevant clinical applications.

## 6. Conclusions

This study demonstrated that high BBB permeability and hypoperfusion statuses derived from CTP parameters were associated with HT. Among the parameters of CTP, relative indices might show better stability and predictive efficacy than absolute indices. RCBF, rPS, and rCBV showed moderate diagnostic performances in the prediction of HT, among which rCBF showed the best predictive efficiency.

## Figures and Tables

**Table 1:** Characteristics of included studies.

Study	Country of Included Patients	Study Design	Time (min) from Onset to Imaging	Number of Patients	Age Mean ± SD/Median (IQR)	Male (%)	CT Scanner Used	Circular Region of Roi	Treatment	Follow-Up Imaging	Hemorrhagic Transformation (n)
Bennink 2015 [21]	Netherlands	Prospective	<9 h	60	69 ± 13	40	40–256	Nr	IVT or EVT	Nr	20
Francesco 2021 [15]	Italy	Prospective	<12 h	171	75.5 ± 11.8	50	128	D = 10 mm	IVT or EVT or both	24–36 h	31
Fu 2012 [12]	China	Retrospective	<8 h	26	62.4 ± 14.21	57.7	16	Nr	Nr	1–7 d	11
Geng 2015 [8]	China	Retrospective	<4.5 h	80	67.5 ± 4.9	66.3	Nr	Nr	IVT or not	7d	38
Huang 2014 [14]	China	Retrospective	<6 h	30	Nr	Nr	8	S = 90 mm^2^	IVT	7d	10
Jain 2012 [16]	America	Retrospective	<12 h	83	72 (61–80)	55.4	64	Nr	Except EVT	Nr	16
Kim 2018 [17]	Korea	Prospective	<9 h	46	66 ± 12	54	64	Nr	IVT or EVT or both	24 h	15
Langel 2019 [18]	Slovenia	Prospective	<4.5 h	75	72.63 ± 11.7	62.7	Nr	D = 15–20 mm	IVT	24 h	35
Li 2017 [23]	China	Retrospective	<6 h	70	Nr	74.3	Nr	Nr	IVT or EVT or not	24 h	14
Li 2020 [11]	China	Retrospective	<24 h	64	Nr	Nr	Nr	Nr	Nr	7 d	22
Lin 2012 [19]	America	Retrospective	<9 h	84	Nr	52.4	16	D = 15 mm	Nr	Nr	22
Richard 2009 [9]	Canada	Prospective	<3 h	41	Nr	63.4	64	Nr	IVT or not	1–5 d	22
Sun 2019 [25]	China	Retrospective	<12 h	200	61.85 ± 6.12	48.5	128	Nr	Nr	Nr	140
Sun 2021 [10]	China	Retrospective	<6 h	58	Nr	55.1	256	D = 15–20 mm	IVT	1–3 d	23
Xiong 2012 [13]	China	Prospective	<9 h	31	65±12	67.7	16	S = 100 mm^2^	IVT	1, 3, 7 d	11
Yen 2016 [20]	Canada	Retrospective	<6 h	42	Nr	42.3	128	Nr	Nr	Nr	15
Zhang 2021 [27]	China	Prospective	<6 h	70	Nr	47.1	256	Nr	EVT	7 d	39
Zuo 2020 [26]	China	Prospective	<12 h	160	62.01 ± 5.98	61.25	64	Nr	Nr	Nr	140

SD = standard deviation; IQR = interquartile range; Nr = not reported; IVT = intravenous thrombolysis; EVT = endovascular thrombectomy; Roi = region of interest; D = diameter; S = square.

**Table 2:** Number of HT subtypes classified according to the ECASS II.

Study	HI1	HI2	HI	PH1	PH2	PH	Any HT
Bennink 2015 [21]	—	—	—	—	—	—	20
Francesco 2021 [15]	—	17	17	4	10	14	31
Fu 2012 [12]	—	—	—	—	—	—	11
Geng 2015 [8]	—	—	—	—	—	—	38
Huang 2014 [14]	—	—	—	—	—	—	10
Jain 2012 [16]	7	6	15	0	1	1	16
Kim 2018 [17]	6	2	8	3	4	7	15
Langel 2019 [18]	—	—	—	—	—	—	35
Li 2017 [23]	—	—	—	2	12	14	14
Li 2020 [11]	—	—	—	—	—	—	22
Lin 2012 [19]	—	—	12	—	—	10	22
Richard 2009 [9]	—	—	15	—	—	8	22
Sun 2019 [25]	—	—	—	—	—	—	140
Sun 2021 [10]	—	—	—	—	—	—	23
Xiong 2012 [13]	—	—	—	—	—	—	11
Yen 2016 [20]	—	—	—	—	—	—	15
Zhang 2021 [27]	—	—	—	—	—	—	39
Zuo 2020 [26]	—	—	—	—	—	—	140

HI = hemorrhagic infarction; PH = parenchymal hematoma; HT = hemorrhagic transformation.

**Table 3:** Characteristics of included studies which provided diagnostic performance of rPS, rCBF, or rCBV.

Study	Reference Standard	rPS	Cutoff rCBF	rCBV
Bennink 2015 [21]	Follow-up NCCT within 3 days or in	1.12	—	—
	case of clinical deterioration			
Fu 2012 [12]	Follow-up NCCT or MRI at 1–7 days	5.81	—	—
Jain 2012 [16]	Follow-up NCCT or MRI	—	—	98%
Langel 2019 [18]	Follow-up NCCT within 24 h	—	4.5%	8.5%
Li 2017 [23]	Any follow-up imaging within 24 h	2.89	—	—
Li 2020 [11]	Follow-up NCCT or within 7 days	2.128	—	—
Sun 2019 [25]	Follow-up NCCT	—	89.2%	48.6%
Sun 2021 [10]	Follow-up NCCT within 3 days	—	23.5%	62.5%
Yen 2016 [20]	Follow-up NCCT	1.3	—	—
Zhang 2021 [27]	Follow-up NCCT or MRI within 7 days	5.6	75.8%	56%
Zuo 2020 [26]	Follow-up NCCT	—	85.6%	41.2%

rPS = relative permeability-surface area product; rCBF = relative cerebral blood flow; rCBV = relative cerebral blood volume; NCCT = non-contrast CT; MRI = magnetic resonance imaging.

**Table 4:** Quality of the 13 included studies that provided the original data of CTP parameters.

Study	A	B	C	D	E	F	G	H	Quality Total/9	Funding Bias
Francesco 2021 [15]	1	1	1	1	1	1	1	1	8	Low
Fu 2012 [12]	1	1	1	1	0	1	1	1	7	Moderate
Geng 2015 [8]	1	1	1	1	1	1	1	1	8	Low
Huang 2014 [14]	1	1	1	1	0	1	1	1	7	Moderate
Jain 2012 [16]	1	1	1	1	2	1	1	0	8	Low
Kim 2018 [17]	1	1	1	1	1	1	0	1	7	Moderate
Langel 2019 [18]	1	1	1	1	0	1	0	1	6	Moderate
Li 2020 [11]	1	1	1	1	0	1	1	1	7	Moderate
Lin 2012 [19]	1	1	1	1	1	1	1	1	8	Low
Richard 2009 [9]	1	1	1	1	1	1	1	1	8	Low
Sun 2021 [10]	1	1	1	1	1	1	1	1	8	Low
Xiong 2012 [13]	1	1	1	1	0	1	1	1	7	Moderate
Yen 2016 [20]	1	1	1	1	1	1	1	0	7	Moderate

NOS analysis criteria: A: Representativeness of the exposed cohort; B: Selection of the non-exposed cohort; C: Ascertainment of exposure; D: Demonstration that outcome of interest was not present at start of study; E: Comparability of cohorts on the basis of the design or analysis; F: Assessment of outcome; G: Was follow-up long enough for outcomes to occur; and H: Adequacy of follow up of cohorts.

**Table 5:** Meta-analysis of CTP parameter values between HT and non-HT groups.

CTP Parameter	Studies Involved	Patients Involved	I^2^ (%)	Random /Fixed Effect Model	MD (95% CI) HT VS. no-HT	*p*-Value	Figure
CBF	5	344	76	Random	−1.57 (−2.98–0.16)	<0.05	Figure 3a
CBV	5	344	83	Random	−0.31 (−0.63–0.00)	<0.05	Figure 3b
PS	5	246	94	Random	1.62 (0.57–2.66)	<0.05	Figure 3c
rMTT	6	307	19	Fixed	0.19 (−0.04–0.43)	>0.05	Figure 4a
rCBF	7	333	39	Fixed	−0.08 (−0.11--0.06)	*p* < 0.00001	Figure 4b
rCBV	6	258	39	Fixed	−0.16 (−0.22–0.10)	*p* < 0.00001	Figure 4c
rPS	4	163	87	Random	2.20 (0.96-3.45)	*p* < 0.001	Figure 4d

## Data Availability

The dataset used and/or analyzed during this study is available from the corresponding author upon reasonable request.
